# Morphology and Function over a One-Year Follow Up Period after Intravitreal Dexamethasone Implant (Ozurdex) in Patients with Diabetic Macular Edema

**DOI:** 10.1371/journal.pone.0145663

**Published:** 2015-12-31

**Authors:** Rodolfo Mastropasqua, Lisa Toto, Enrico Borrelli, Luca Di Antonio, Chiara De Nicola, Alessandra Mastrocola, Marta Di Nicola, Paolo Carpineto

**Affiliations:** 1 Ophthalmology Clinic, University of Verona, Verona, Italy; 2 Department of Medicine and Science of Aging, Ophthalmology Clinic, University "G. d'Annunzio" Chieti-Pescara, Chieti, Italy; 3 Center of Excellence on Aging and Department of Biomedical Science, University "G. D’Annunzio" of Chieti, Laboratory of Biostatistics, Chieti, Italy; University of Melbourne, AUSTRALIA

## Abstract

**Background:**

To investigate changes in macular morphology and function after an intravitreal dexamethasone implant for diabetic macular edema (DME).

**Methods:**

Twenty-seven eyes in 27 treatment-naive patients affected by DME were treated with intravitreal Ozurdex® injections (IVOI) and followed up 12 months to evaluate morphological and functional changes by means of best-corrected visual acuity (BCVA), microperimetry (MP1), multifocal electroretinography (mfERG), pattern electroretinography (PERG) and spectral domain optical coherence tomography (SD-OCT).

**Results:**

Both BCVA and retinal sensitivity improved significantly at one month after the IVOI (p = 0.031 and p<0.0001, respectively). After five months, the improvement of BCVA remained statistically significant compared with baseline values (p = 0.022); retinal sensitivity improvement was statistically significant for up to four months after the IVOI (p = 0.059). Moreover, central macular thickness significantly decreased for up to four months. Interestingly, PERG and mfERG values did not change significantly for up to four months post-IVOI, but then began to worsen.

**Conclusions:**

In eyes with DME, intravitreal dexamethasone implant determined morphological and functional improvement as soon as one month and for up to four months after the treatment.

## Introduction

Diabetic macular edema (DME), common in patients with diabetes, results from a blood-retinal barrier defect that leads to vascular leakage, fluid accumulation and macula thickening [1]. This breakdown is the result of the expression of inflammatory factors [2], including vascular endothelial growth factor (VEGF), intercellular adhesion molecule-1, interleukin-6, monocyte chemotactic protein-1 and leukostasis [3].

Standard care for DME includes medical control of diabetes (monitoring glycemia and glycated hemoglobin (HbA1c) levels) and focal/grid laser photocoagulation of leaking microaneurysms and areas of diffuse capillary bed leakage [4]. Over the past decade, advances in elucidating the pathogenesis of DME has led to new therapies, including anti-vascular endothelial growth factor (VEGF) agents and corticosteroids. The anti-VEGF agents were the first to be approved for DME treatment[5]. Nevertheless, not all patients respond to the treatment and the compliance to the treatment is not high because of the numerous required injections during the year [6,7].

In 2014, a sustained-release intravitreal 0.7 mg dexamethasone delivery system was approved by Food and Drug Administration (FDA) and Commission Européenne (CE) for the treatment of DME, based on the MEAD study results [8]. Intravitreal corticosteroids block production of inflammatory mediators, such as VEGF, and inhibit leukostasis [9,10]. Dexamethasone is an anti-inflammatory agent that is six-times more active than triamcinolone and 30-times more than cortisol (MEAD study). Furthermore, Boyer et al. reported its efficacy and safety in the treatment of DME when delivered to the vitreous cavity by a sustained-release intravitreal implant [8]. Indeed, studies showed an improvement in central macular thickness (CMT) and best corrected visual acuity (BCVA) in patients affected by DME after treatment [11–14]. Nevertheless, no study has assessed functional changes after treatment by means of microperimetry and electro-functional tests.

In this prospective study, we investigated the changes in macular morphology and function by means of spectral-domain optical coherence tomography (SD-OCT), microperimetry (MP1), multifocal electroretinogram (mfERG) and pattern electroretinography (PERG) in eyes undergoing an intravitreal dexamethasone implant (Ozurdex) for DME over a 12-month period.

## Methods

### Study participants

Twenty-seven type 2 diabetic patients were enrolled for the study; all patients were intravitreal treatment-naive and were affected by a single eye with decreased VA that was secondary to DME. Patients consecutively presented at the University Gabriele D’Annunzio Department of Ophthalmology, between January 2014 and March 2014. This study was approved by our Institutional Review Board “Department of Medicine and Science of Aging, University "G. d'Annunzio" Chieti-Pescara, Italy”, and patients signed informed consent for the use of their data. The study adhered to the tenets of the Declaration of Helsinki.

Criteria for inclusion were: 1) age >18 years old, 2) presence of DME, 3) best-corrected visual acuity (BCVA) of at least 1.0 LogMAR in the study eye at baseline examination (to ensure proper execution of functional examination) and 4) central macular thickness (CMT) >300 μm as measured by spectral-domain optical coherence tomography (SD-OCT) at baseline examination.

The exclusion criteria were 1) structural damage within a 0.5 disc diameter of the center of the macula in the study eye to not preclude improvement in visual acuity following the resolution of macular edema, including atrophy of the retinal pigment epithelium, subretinal fibrosis, laser scar(s), epiretinal membrane involving fovea or organized hard exudative plaques, 2) any ocular surgery and/or laser treatment in the study eye in the last six months, 3) previous intravitreal injection of corticosteroids or anti-VEGF, 4) a history of ocular inflammation or 5) history of IOP elevation in response to steroid treatment or a history of glaucoma.

### Study Protocol

At baseline, all patients underwent a complete ophthalmic evaluation, including assessment of BCVA using Early Treatment Diabetic Retinopathy Study (ETDRS) charts, tonometry, slit-lamp biomicroscopy, indirect fundus ophthalmoscopy and SD-OCT with automated CMT measurements. Furthermore, all patients were tested by using microperimetry, mfERG and PERG to evaluate macula’s function.

All patients were treated with a sustained-release dexamethasone 0.7 mg intravitreal implant (DEX implant; Ozurdex, Allergan Inc., Irvine, CA) within 3±2 days from baseline examination. All injections were performed in the operation room, and the DEX implant was inserted into the vitreous cavity through the pars plana using a customized, single-use 22-gauge applicator. Patients were treated with a topical ophthalmic antibiotic for 10 days after the treatment.

Starting from month 3, in patients with a loss of five letters in BCVA and recurrence/persistence of ME as documented by indirect fundus ophthalmoscopy and SD-OCT (CMT > 300 μm), the treating physicians were free to decide whether to re-administer an intravitreal dexamethasone implant.

Outcome measures included the mean change in BCVA, CMT, microperimetry, mfERG and PERG values from baseline to months 1, 2, 3, 4, 5, 6 and 12. Moreover, we compared the mean change that was expressed as a percentage of BCVA, CMT, microperimetry, mfERG and PERG from baseline to four months after the last injection independent of primary injection or retreatment injection.

In addition, mean values of functional and morphological parameters were compared between patients requiring one injection (group 1) and patients treated with more than one injection (group 2) at the end of the follow-up period.

The number of intravitreal injections and the average period between injections were evaluated during the follow-up period.

The incidence of side effects following DEX implant, injections was recorded for each patient.

### Procedures

SD-OCT (Cirrus HD-OCT, software version 6.0; Carl Zeiss Meditec, Inc., Dublin, CA, USA) was performed to obtain automated CMT measurements that were generated by means of the Macular Cube 512x128 protocol.

Microperimetry was performed using the MP1 Microperimeter (Nidek Technologies, Padova, Italy). The examination was performed using a fixation target consisting of a red cross that was 2° in diameter on a white monochromatic background with a stimulus size Goldmann III projected for 200 msec. A grid of 68 stimuli (Humphrey 10–2) that were randomly presented was applied to retinal locations covering the central 10°.

Pattern electroretinograms (PERG) and multifocal ERGs (Retimax CSO, Florence, Italy) were recorded for each patient, according to the International Society for Clinical Electrophysiology of Vision (ISCEV) protocols that were updated in 2012 and 2011[15,16]. The PERG waveform in normal subjects usually consists of a small initial negative component with a peak time of approximately 35 ms (N35), followed by a much larger positive component at 45–60 ms (P50). This positive component is followed by a large negative component at 90–100 ms (N95). Concerning mfERG, the ocular fundus was segmented by an array of 61 hexagons, and average responses for the implicit times and amplitudes of N1 (first negative component) and P1 (first positive component) of the first-order kernel were calculated for two regional ring groups (R1 and R2).

### Statistical analysis

Statistical calculations were performed using Statistical Package for Social Sciences (version 20.0. SPSS Inc. Chicago. IL. USA). To detect departures from normality distribution, Shapiro-Wilk’s test was performed for all variables. All quantitative variables were presented as median and interquartile range (IQR) in the results and in the tables.

Friedman test was applied to compare the mean BCVA, sensitivity at microperimetry, N1 and P1 amplitudes at mfERG, P50 and N95 amplitude and latency at PERG, CMT during the follow-up. Wilcoxon signed-rank test with correction for multiple comparison was applied to compare baseline values with each follow-up controls. Comparisons at 12 months between the group undergoing only one treatment and the group undergoing two treatments were performed using Mann-Whitney U test. Moreover, we compared the mean change expressed as a percentage of morphological and functional parameters (BCVA. CMT. microperimetry. mfERG and PERG) from baseline to four months after the last injection, independent of primary injection or retreatment injection, using Wilcoxon signed-rank test.

Non-parametric correlation analysis (Spearman test) was conducted to determine the relationship between BCVA, MP1 and OCT.

## Results

Twenty-seven eyes of 27 type 2 diabetic patients (21 males; mean age 66.44±11.56 years), affected by DME and undergoing a dexamethasone implant treatment, were included for the analysis. At 12 months, nine out of 27 eyes completed the follow-up with only one intravitreal dexamethasone implant treatment; all other eyes received a second implant during the follow-up period. The average period between the first and second treatments was 6.7±2.3 months.

The mean duration of diabetes was 15.83±12.56 years. The mean duration of DME was 5.72±1.87 months.

One month after an intravitreal dexamethasone implant, the BCVA significantly improved from 0.33 (0.19 to 0.37) LogMAR (baseline) to 0.31 (0.17 to 0.37) LogMAR (p = 0.031). The BCVA was 0.27 (0.15 to 0.34), 0.11 (0.10 to 0.21) and 0.13 (0.05 to 0.21) LogMAR at two, three and four months after the treatment, respectively (p = 0.037, p<0.0001 and p = 0.001 after the comparison with baseline values, respectively) (Table 1).

**Table 1:** Functional and morphological parameters evaluated at each time-point from intravitreal dexamethasone implant.

Diabetic patients								
(n = 27)								
		Baseline	1 month	2 month	3 month	4 month	5 month	6 month
		(n = 27)	(n = 27)	(n = 27)	(n = 27)	(n = 27)	(n = 21)	(n = 12)
***BCVA (LogMAR)***		0.33 (0.19 to 0.39)	0.31 (0.17 to 0.37)	0.27 (0.15 to 0.34)	0.11 (0.10 to 0.21)	0.13 (0.05 to 0.21)	0.11 (-0.01 to 0.18)	0.12 (0.01 to 0.51)
			p = 0.031	p = 0.037	p<0.0001	p = 0.001	p = 0.022	p = 0.236
***MP1 10*** ^***o***^ ***sensitivity (dB)***		7.55 (7.10 to 8.55)	11.50 (10.70 to 14.40)	9.70 (8.67 to 11.02)	9.54 (8.65 to 10.83)	9.05 (8.50 to 10.53)	9.15 (5.65 to 12.05)	9.01 (5.14 to 11.04)
			p<0.0001	p<0.0001	p = 0.003	p = 0.001	p = 0.059	p = 0.155
***mfERG***								
	***P1R1 amplitude (μV)***	0.88 (0.76 to 0.93)	0.64 (0.47 to 0.68)	0.68 (0.54 to 0.75)	0.68 (0.42 to 0.80)	0.58 (0.47 to 0.85)	0.51 (0.32 to 0.83)	0.50 (0.35 to 0.79)
			p = 0.093	p = 0.117	p = 0.131	p = 0.224	p = 0.041	p = 0.037
	***N1R1 amplitude (μV)***	-0.50 (-0.68 to -0.38)	-0.20 (-0.33 to 0.00)	-0.20 (-0.33 to 0.15)	-0.46 (-0.68 to -0.10)	-0.45 (-0.68 to 0.08)	-0.11 (-0.21 to 0.08)	-0.32 (-0.50 to 0.07)
			p = 0.213	p = 0.197	p = 0.112	p = 0.6	p = 0.342	p = 0.477
	***P1R2 amplitude (μV)***	0.42 (0.32 to 0.55)	0.47 (0.29 to 0.50)	0.47 (0.27 to 0.50)	0.38 (0.33 to 0.47)	0.33 (0.32 to 0.47)	0.31 (0.21 to 0.46)	0.31 (0.21 to 0.47)
			p = 0.406	p = 0.406	p = 0.828	p = 0.564	p = 0.077	p = 0.058
	***N1R2 amplitude (μV)***	-0.24 (-0.27 to -0.16)	-0.29 (-0.31 to -0.22)	-0.29 (-0.31 to 0.10)	-0.23 (-0.39 to -0.13)	-0.13 (-0.29 to -0.12)	-0.29 (-0.35 to -0.22)	-0.22 (-0.35 to 0.02)
			p = 0.885	p = 0.312	p = 0.828	p = 0.090	p = 0.565	p = 0.477
***PERG***								
	***P50-N95 amplitude (μV)***	2.12 (1.36 to 3.77)	2.05 (1.40 to 2.57)	2.04 (1.29 to 2.99)	2.12 (1.75 to 2.15)	1.91 (1.74 to 2.00)	1.35 (0.57 to 3.02)	1.20 (0.64 to 2.88)
			p = 0.279	p = 0.385	p = 0.885	p = 0.270	p = 0.037	p<0.0001
	***P50 latency (msec)***	55.42 (50.29 to 60.23)	55.42 (51.51 to 60.55)	57.13 (53.22 to 57.62)	57.37 (52.73 to 64.70)	62.11 (60.06 to 70.92)	64.15 (60.06 to 68.08)	63.47 (63.20 to 67.22)
			p = 0.470	p = 0.606	p = 0.564	p = 0.065	p = 0.008	p = 0.008
***CMT (μm)***		358 (331 to 558)	279 (254 to 334)	253 (228 to 294)	284 (233 to 299)	287 (233 to 327)	300 (292 to 394)	300 (299 to 450)
			p<0.0001	p<0.0001	p<0.0001	p<0.0001	p = 0.13	p = 0.236

**BCVA:** best-corrected visual acuity (logMAR [logarithm of the minimum angle of resolution]); **MP1:** microperimetry; **CMT:** central macular thickness; **n**: number of patients. Data are presented as median (Interquartile Range, IQR). The Wilcoxon signed-rank test was performed in order to obtain p-values for the parameters’ comparison between baseline and each follow-up visit.

**Fig 1:**
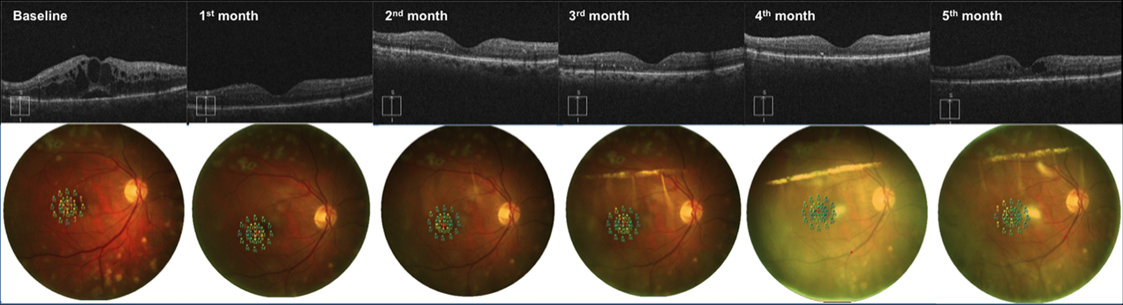
Follow-up of a patient affected by diabetic macular edema undergoing Ozurdex implant. Optical coherence tomography scans and MP1 microperimetry outputs, from a 62-year-old patient affected by diabetic macular edema in his right eye, acquired at baseline visit, 1 month, 2 months, 3 months, 4 months and 5 months after the first Ozurdex injection. The patient underwent a second Ozurdex implant at 5^th^ month because of edema reappearance.

Similarly, the retinal sensitivity in microperimetry improved from 7.55 (7.10 to 8.55) dB (baseline) to 11.50 (10.70 to 14.40) dB at one month (p<0.0001). Moreover, the retinal sensitivity was 9.70 (8.67 to 11.02) dB, 9.54 (8.65 to 10.83) dB and 9.05 (8.50 to 10.53) dB at two, three and four months after the treatment, respectively, (p<0.0001, p = 0.003 and p = 0.001, respectively, compared with the baseline value) (Table 1, Fig 1).

The CMT decreased from 358 (331 to 558) μm (baseline) to 279 (254 to 334) μm (one month postoperatively) (p<0.0001). At two, three and four months, the CMT was 253 (228 to 294) μm, 284 (233 to 299) μm and 287 (233 to 327) μm, respectively (p<0.0001 in all the three comparisons with the baseline value) (Table 1, Fig 1).

At four months, attending physicians decided to perform retreatment with an intravitreal dexamethasone implant in six eyes; then, only 21 eyes were included in the five-month analysis.

At five months, an additional nine eyes underwent retreatment; therefore, 12 eyes were included in the six-month analysis.

The BCVA remained significantly improved five months after the treatment compared with baseline values (median: 0.11 LogMAR; IQR: -0.01 to 0.18 LogMAR; p = 0.022); the improvement was not present after six months (median: 0.12 LogMAR; IQR: 0.01 to 0.51 LogMAR; p = 0.236).

At five and six months after the treatment, the improvement of retinal sensitivity at MP1 was not still significant compared with baseline values (median: 9.15 dB and 9.01 dB; IQR: 5.65 to 12.05 dB and 5.14 to 11.04 dB; p = 0.059 and p = 0.155; respectively).

The CMT decrease was not statistically significant at five and six months after the implant (median: 300 μm and 300 μm; IQR: 292 to 394 μm and 299 to 450 μm; p = 0.13 and p = 0.236; respectively).

mfERG N1R1 and P1R1 amplitudes, as well as N1R2 and P1R2 amplitudes, did not change significantly from administration of dexamethasone up to the fourth month of follow-up (Table 1). Nevertheless, P1R1 amplitude decreased slightly five and six months after the injection (median: 0.88 μV and IQR: 0.76 to 0.93 at baseline; median: 0.51 μV and IQR: 0.32 to 0.83 μV at five months; median: 0.50 μV and IQR: 0.35 to 0.79 at six months; p = 0.041 and p = 0.037, respectively).

PERG P50 amplitude increased, but not significantly, up to the fourth month of follow-up and decreased after five months (median: 2.12 μV and IQR: 1.36 to 3.77 μV at baseline; median: 1.35 μV, IQR: 0.57 to 3.02 μV and p<0.0001 at five months; median: 1.20 μV, IQR: 0.64 to 2.88 μV and p = 0.037 at six months) (Table 1). PERG P50 latency (median: 55.42 msec and IQR: 50.29 to 60.23 msec at baseline) did not change up to the fourth month of follow-up; however, it significantly increased at five and six months (median: 64.15 msec, IQR: 60.06 to 68.08 msec and p = 0.008 at 5 months; median: 63.47 msec, IQR: 63.20 to 67.22 msec and p = 0.008 at 6^th^ month) (Table 1). Interestingly, eyes that did not undergo retreatment within 12 months from the first dexamethasone implant showed a better retinal sensitivity at baseline, compared with eyes undergoing retreatment <12 months (median: 8.8 dB and IQR: 7.6 to 9.4 dB vs median: 7.8 dB and IQR: 4.5 to 7.9 dB; p = 0.020).

At 12 months, no difference was found between patients undergoing one treatment and patients treated with 2 dexamethasone implants (Table 2).

**Table 2:** Comparison of functional and morphological parameters, evaluated at 12 months after intravitreal dexamethasone implant, between patients undergoing one treatment and patients undergoing two treatments.

Diabetic patients				
(n = 27)				
		12 month (1 IV injection, n = 9)	12 month (2 IV injection, n = 18)	*p-value*
***BCVA (LogMAR)***		0.14 (-0.02 to 0.26)	0.21 (0.14 to 0.30)	0.253
***MP1 10*** ^***o***^ ***sensitivity (dB)***		10.40 (10.00 to 11.00)	9.55 (9.10 to 14.50)	0.176
***mfERG***				
	***P1R1 amplitude (μV)***	0.75 (0.56 to 0.88)	0.77 (0.77 to 0.85)	0.633
	***N1R1 amplitude (μV)***	-0.30 (-0.98 to -0.26)	-0.30 (-0.34 to -0.30)	0.806
	***P1R2 amplitude (μV)***	0.68 (0.39 to 1.19)	0.67 (0.61 to 0.70)	0.326
	***N1R2 amplitude (μV)***	-0.09 (-0.59 to 0.00)	-0.30 (-0.30 to 0.27)	0.152
***PERG***				
	***P50-N95 amplitude (μV)***	1.70 (1.28 to 3.97)	1.19 (1.11 to 1.55)	0.112
	***P50 latency (msec)***	62.99 (57.28 to 65.66)	64.32 (58.28 to 64.32)	0.879
***CMT (μm)***		301 (299 to 312)	316 (311 to 327)	0.176

**BCVA:** best-corrected visual acuity (logMAR [logarithm of the minimum angle of resolution]); **MP1:** microperimetry; **CMT:** central macular thickness; **n**: number of patients. Data are presented as median (Interquartile Range, IQR). The Mann-Whitney U test was performed in order to obtain p-values.

Furthermore, within group 2, we calculated the variation expressed as a percentage of BCVA, MP1 and CMT between baseline and four-month control after the treatment with either the first treatment or the retreatment. No statistically significant differences were found between the latter two (Table 3).

**Table 3:** Comparison of effectiveness between first and second treatment.

Diabetic patients undergoing 2 treatments			
(n = 21)			
	First treatment	Second treatment	*p-value*
***BCVA (LogMAR)***	-52.5% (-65.0% to -43.0%)	-11.5% (-35.0% to +38.0%)	0.04
***MP1 10*** ^***o***^ ***sensitivity (dB)***	+64.0% (+12.0% to +79.0%)	+21.5% (-15.0% to +127%)	0.743
***CMT (μm)***	-18.0% (-36.0% to -4.0%)	-22.0% (-37.0% to -4.0%)	0.212

**BCVA:** best-corrected visual acuity (logMAR [logarithm of the minimum angle of resolution]); **MP1:** microperimetry; **CMT:** central macular thickness; **n**: number of patients. Data are delta percentages (median and Interquartile Range, IQR) after 4 months from the treatment. The Mann-Whitney U test was performed in order to obtain p-values.

Moreover, we evaluated the correlation among CMT, BCVA and MP1 values. We found an inverse correlation between MP1 and BCVA values (then an improvement in MP1 is correlated with an improvement in BCVA values) that did not reach the statistical significance only at the 4^th^ month. Furthermore, CMT values were directly correlated with BCVA values and inversely correlated with MP1 values, in almost all the months (Table 4).

**Table 4:** Correlation analysis at each time-point among functional and morphological parameters.

		Baseline			1 Month			2 Months			3 Months			4 Months			5 Months			6 Months		
		n = 27			n = 27			n = 27			n = 27			n = 27			n = 21			n = 12		
		*CMT*	*MP1*	*BCVA*	*CMT*	*MP1*	*BCVA*	*CMT*	*MP1*	*BCVA*	*CMT*	*MP1*	*BCVA*	*CMT*	*MP1*	*BCVA*	*CMT*	*MP1*	*BCVA*	*CMT*	*MP1*	*BCVA*
***BCVA***	ρ	0.701	-0.646	-	0.481	-0.789	-	0.197	-0.768	-	0.323	-0.421	-	0.359	-0.294	-	0.327	-0.616	-	0.958	-0.988	-
	p	<0.0001	<0.0001	-	0.011	<0.0001	-	0.324	<0.0001	-	0.178	0.033	-	0.125	0.137	-	0.147	0.003	-	<0.0001	<0.0001	-
***MP1***	ρ	-0.447	-	-0.646	-0.254	-	-0.789	-0.497	-	-0.768	-0.334	-	-0.421	0.246	-	-0.294	-0.320	-	-0.616	-0.923	-	-0.988
	p	0.019	-	<0.0001	0.2	-	<0.0001	0.008	-	<0.0001	0.074	-	0.033	0.217	-	0.137	0.157	-	0.003	<0.0001	-	<0.0001
***CMTT***	ρ	-	-0.447	0.701	-	-0.254	0.481	-	-0.497	0.197	-	-0.334	0.323	-	0.246	0.359	-	-0.320	0.327	-	-0.923	0.958
	p	-	0.019	<0.0001	-	0.2	0.011	-	0.008	0.324	-	0.074	0.178	-	0.217	0.125	-	0.157	0.147	-	<0.0001	<0.0001

**BCVA:** best-corrected visual acuity; **MP1:** microperimetry; **CMT:** central macular thickness; **n**: number of patients. Non-parametric correlation analysis (Spearman test) was conducted in order to obtain p-values.

No IOP elevation >21 mmHg or cataract progression were observed during the study follow-up.

## Discussion

In this prospective study, we investigated changes in function and macular morphology in eyes undergoing DEX implants for diabetic macular edema, over a 12-month follow-up period. Overall, we found that this treatment induced an improvement in BCVA and macular sensitivity as soon as one month after administration, and this effect persisted for up to five months. CMT showed a similar trend; it significantly decreased as soon as one month after the implant and was still reduced four months after treatment.

Over the past decade, several studies demonstrated efficacy and safety of the DEX implant in patients affected by DME[8,11–13,17]. This led to treatment approval in 2014. Our results were in according with these studies [8,11–13] showing a sustained BCVA and CMT improvement in eyes undergoing DEX treatment without the development of serious side effects. Nevertheless, to the best of our knowledge, no other report about the functional evaluation of the efficacy of DEX implants in DME exists.

Microperimetry has a high sensitivity in evaluating retinal function in diabetic patients[18] and is very useful in estimating visual prognosis in DME [19]. Our data showed an improvement of retinal sensitivity in the first five months after DEX implant.

Moreover, Boarse et al. supported the role of electro-functional assessments in diabetes, showing a compromise in mfERG and PERG values in the early stages of diabetic retinopathy in patients affected by DME [20]. Electro-functional alterations are secondary to neuroretinal degeneration affecting diabetic patients [20]. Our results did not show an improvement of electro-functional values following a DEX implant. Nevertheless, the values were stable for up to four months, worsening only after the fifth month. Interestingly, Querques et al. reported that electro-functional values do not improve also after DEX implants in other forms of chronic macular edema, as opposed to improvement of MP1 values[21,22]. Then, the results of the present study suggest that treatment led to both a reduction of edema and to stabilization of neuroretinal degeneration; the latter restarts when the treatment is no longer effective.

Several studies reported there is a close link between inflammation, diabetic macular edema and neurodegeneration[23,24]. Indeed, some authors showed that high pro-inflammatory cytokines being involved in the pathogenesis of macular edema and that inflammatory cytokines and chemokines can be detected in the aqueous humor of patients affected by DME[25]. Moreover, it is known that pro-inflammatory factors play an important role in the retina neurodegeneration[24]. There are several theories about the relationship between inflammation and neurodegeneration: (i) the leukostasis could create areas of capillary nonperfusion within the retina, leading to neuroretinal ischemia[3]; (ii) TNF-alpha, a cytokine over-expressed in diabetes, could led to retinal neuron apoptosis[26]. Then, we suggested that dexamethasone implant could reduce or slow down the neurodegeneration by reducing the inflammation.

Starting from the third month, treating physicians were free to decide whether to re-administer an intravitreal dexamethasone implant on the basis of combined functional and morphological findings. During the 12-month study period, 18 out of 27 patients benefited from one retreatment after the first intravitreal dexamethasone implant. Furthermore, 15 out of 18 eyes needed retreatment before the sixth month. Boyer et al. recently reported the results of the MEAD trial [8] showing functional and anatomical outcomes comparable to ours. The MEAD study protocol defined six months as the minimum interval time between two treatments. Nevertheless, several studies recently showed that the maximum interval before retreatment should be less than six months [27]. Indeed, our functional and morphological results suggest the latter aspect. In particular, considering its importance in diabetic retinopathy pathogenesis and visual prognosis, we believe that it is important to block neuroretinal degeneration, which occurs in the first four months after treatment.

Interestingly, at 12 months, the 18 eyes undergoing two treatments and the 9 eyes undergoing 1 treatment did not show difference in morphological and functional values. The latter data suggest that the decision of not treating patients with a durable morphological and functional response to the treatment does not worsen the prognosis.

Moreover, we compared the effectiveness of the first treatment with retreatment. Recently, Scaramuzzi et al.[27] showed a sustained functional (BCVA) and morphological (CMT) improvement in eyes affected by DME after repeated injection. Our data partially confirmed these results (improvement in BCVA was higher in the treatment compared to the retreatment) and, in addition, showed a sustained retinal sensitivity improvement after the retreatment.

The design of the current study also allowed analysis, in intravitreal treatment-naïve eyes with DME, the relationship among morphological and functional parameters. An inverse correlation was found between CMT and retinal sensitivity in almost all eyes during the first six months after treatment. The latter finding confirms the importance of reducing edema to improve retinal function.

Our study has several limitations. The series presented here is relatively small. The latter aspect, in addition to the fact that this study lacks a control group, implies that differences during the follow-up could have been occurred even if the treatment was not applied. However, one should look at the current series in consideration of the strict inclusion criteria, especially the fact that we enrolled only treatment naïve patients. Finally, because our patients were not randomized, some conclusions, such as the period between the treatment and the re-treatment, are only suggested.

In conclusion, we showed that in eyes with DME, intravitreal dexamethasone implants provide meaningful functional benefits as soon as one month after treatment. Current findings relying on microperimetry and electro-functional exam changes further demonstrated that DEX implant improved retinal function and blocked neuroretinal degeneration for as long as the DEX implant worked. This study raises many questions and prompts further investigation including: (i) a randomized controlled trial in order to confirm many of our conclusions; (ii) the evaluation of new therapeutic approaches targeted to reduce inflammation and/or neuroretinal degeneration; (iii) the assessment of the effect of VEGF antagonists on electro-functional exams in eyes affected by DME; and (iv) the test of the consequences of DEX implant in patients already treated with VEGF antagonists.

## References

[1] Bhagat N, Grigorian RA, Tutela A, Zarbin MA. Diabetic macular edema: pathogenesis and treatment. Surv Ophthalmol;54:1–32. 10.1016/j.survophthal.2008.10.001 19171208

[2] Funatsu H, Noma H, Mimura T, Eguchi S, Hori S. Association of Vitreous Inflammatory Factors with Diabetic Macular Edema. Ophthalmology 2009;116:73–79. 10.1016/j.ophtha.2008.09.037 19118698

[3] Miyamoto K, Khosrof S, Bursell SE, Rohan R, Murata T, Clermont AC, et al Prevention of leukostasis and vascular leakage in streptozotocin-induced diabetic retinopathy via intercellular adhesion molecule-1 inhibition. Proc Natl Acad Sci U S A 1999;96:10836–41. 10485912 10.1073/pnas.96.19.10836 PMC17969

[4] Zitkus BS. Update on the American Diabetes Association Standards of Medical Care. Nurse Pract 2014;39:22–32. 10.1097/01.NPR.0000451880.48790.50 24979246

[5] Pacella E, La Torre G, Impallara D, Malarska K, Turchetti P, Brillante C, et al Efficacy and safety of the intravitreal treatment of diabetic macular edema with pegaptanib: a 12-month follow-up. Clin Ter 2013;164:e121–6. 10.7417/CT.2013.1543 23698213

[6] Nguyen QD, Brown DM, Marcus DM, Boyer DS, Patel S, Feiner L, et al Ranibizumab for Diabetic Macular Edema. Ophthalmology 2012;119:789–801. 10.1016/j.ophtha.2011.12.039 22330964

[7] Brown DM, Nguyen QD, Marcus DM, Boyer DS, Patel S, Feiner L, et al Long-term Outcomes of Ranibizumab Therapy for Diabetic Macular Edema: The 36-Month Results from Two Phase III Trials. Ophthalmology 2013;120:2013–2022. 10.1016/j.ophtha.2013.02.034 23706949

[8] Boyer DS, Yoon YH, Belfort R, Bandello F, Maturi RK, Augustin AJ, et al Three-year, randomized, sham-controlled trial of dexamethasone intravitreal implant in patients with diabetic macular edema. Ophthalmology 2014;121:1904–14. 10.1016/j.ophtha.2014.04.024 24907062

[9] Wang K, Wang Y, Gao L, Li X, Li M, Guo J. Dexamethasone inhibits leukocyte accumulation and vascular permeability in retina of streptozotocin-induced diabetic rats via reducing vascular endothelial growth factor and intercellular adhesion molecule-1 expression. Biol Pharm Bull 2008;31:1541–6. 18670086 10.1248/bpb.31.1541

[10] Tamura H, Miyamoto K, Kiryu J, Miyahara S, Katsuta H, Hirose F, et al Intravitreal injection of corticosteroid attenuates leukostasis and vascular leakage in experimental diabetic retina. Invest Ophthalmol Vis Sci 2005;46:1440–4. 15790913 10.1167/iovs.04-0905

[11] Bandello F, Lattanzio R, Zarbin MA, Zucchiatti I. Clinical Strategies in the Management of Diabetic Retinopathy: A Step-by-Step Guide for Ophthalmologists. 1st ed. Springer-Verlag Berlin Heidelberg;2014.

[12] Haller JA. Randomized Controlled Trial of an Intravitreous Dexamethasone Drug Delivery System in Patients With Diabetic Macular Edema. Arch Ophthalmol 2010;128:289 10.1001/archophthalmol.2010.21 20212197

[13] Bandello F, Battaglia Parodi M, Tremolada G, Lattanzio R, De Benedetto U, Iacono P. Steroids as part of combination treatment: the future for the management of macular edema? Ophthalmologica 2010;224 Suppl:41–5. 20714180 10.1159/000315161

[14] Pacella E, Vestri AR, Muscella R, Carbotti MR, Castellucci M, Coi L, et al Preliminary results of an intravitreal dexamethasone implant (Ozurdex®) in patients with persistent diabetic macular edema. Clin Ophthalmol 2013;7:1423–8. 10.2147/OPTH.S48364 23901252 PMC3720664

[15] Hood DC, Bach M, Brigell M, Keating D, Kondo M, Lyons JS, et al ISCEV standard for clinical multifocal electroretinography (mfERG) (2011 edition). Doc Ophthalmol 2012;124:1–13. 10.1007/s10633-011-9296-8 PMC4466109 22038576

[16] Bach M, Brigell MG, Hawlina M, Holder GE, Johnson MA, McCulloch DL, et al ISCEV standard for clinical pattern electroretinography (PERG): 2012 update. Doc Ophthalmol 2012;126:1–7. 10.1007/s10633-012-9353-y 23073702

[17] Pacella E, La Torre G, Turchetti P, Merisola C, Lenzi T, Mazzeo F, et al Evaluation of efficacy dexamethasone intravitreal implant compared to treatment with anti-VEGF in the treatment of diabetic macular edema. Senses Sci 2014;1(4):164–168.

[18] De Benedetto U, Querques G, Lattanzio R, Borrelli E, Triolo G, Maestranzi G, et al Macular dysfunction is common in both type 1 and type 2 diabetic patients without macular edema. Retina 2014;34:2171–7. 10.1097/IAE.0000000000000205 24978668

[19] Vujosevic S, Midena E, Pilotto E, Radin PP, Chiesa L, Cavarzeran F. Diabetic macular edema: correlation between microperimetry and optical coherence tomography findings. Invest Ophthalmol Vis Sci 2006;47:3044–51. 16799051 10.1167/iovs.05-1141

[20] Bearse MA, Ozawa GY. Multifocal Electroretinography in Diabetic Retinopathy and Diabetic Macular Edema. Curr Diab Rep. 2014;14:526 10.1007/s11892-014-0526-9 25005120

[21] Querques G, Cascavilla ML, Cavallero E, Triolo G, Querques L, Lattanzio R, et al Changes in macular function after ozurdex for retinal vein occlusion. Optom Vis Sci 2014;91:760–8. 10.1097/OPX.0000000000000308 24927143

[22] Querques G, Lattanzio R, Querques L, Triolo G, Cascavilla ML, Cavallero E, et al Impact of intravitreal dexamethasone implant (Ozurdex) on macular morphology and function. Retina 2014;34:330–41. 10.1097/IAE.0b013e31829f7495 23945638

[23] Simó R, Hernández C. Neurodegeneration in the diabetic eye: new insights and therapeutic perspectives. Trends Endocrinol Metab 2014;25:23–33. 10.1016/j.tem.2013.09.005 24183659

[24] Stem MS, Gardner TW. Neurodegeneration in the pathogenesis of diabetic retinopathy: molecular mechanisms and therapeutic implications. Curr Med Chem 2013;20: 3241–50. 23745549 10.2174/09298673113209990027 PMC4071765

[25] Dong N, Xu B, Chu L, Tang X. Study of 27 Aqueous Humor Cytokines in Type 2 Diabetic Patients with or without Macular Edema. PLoS One 2015;10:e0125329 10.1371/journal.pone.0125329 25923230 PMC4414516

[26] Huang H, Gandhi JK, Zhong X, Wei Y, Gong J, Duh EJ, et al TNFalpha is required for late BRB breakdown in diabetic retinopathy, and its inhibition prevents leukostasis and protects vessels and neurons from apoptosis. Invest Ophthalmol Vis Sci 2011;52:1336–44. 10.1167/iovs.10-5768 21212173 PMC3101693

[27] Scaramuzzi M, Querques G, Spina C La Lattanzio R, Bandello F. Repeated intravitreal dexamethasone implant (Ozurdex) for diabetic macular edema. Retina 2015;35:1216–22. 10.1097/IAE.0000000000000443 25574787

